# Effective combination of cold physical plasma and chemotherapy against Ewing sarcoma cells in vitro

**DOI:** 10.1038/s41598-024-56985-4

**Published:** 2024-03-18

**Authors:** Andreas Nitsch, Sara Qarqash, Sarah Römer, Janosch Schoon, Debora Singer, Sander Bekeschus, Axel Ekkernkamp, Georgi I. Wassilew, Mladen V. Tzvetkov, Lyubomir Haralambiev

**Affiliations:** 1 Center for Orthopedics, Trauma Surgery and Rehabilitation Medicine, University Medicine Greifswald, Ferdinand-Sauerbruch-Straße, 17475 Greifswald, Germany; 2 Department of General Pharmacology, Institute of Pharmacology, Center of Drug Absorption and Transport (C_DAT), University Medicine Greifswald, Felix-Hausdorff-Straße 3, 17489 Greifswald, Germany; 3 Clinic and Polyclinic for Dermatology and Venerology, Strempelstr. 13, 18057 Rostock, Germany; 4 Leibniz Institute for Plasma Science and Technology (INP), ZIK Plasmatis, Felix-Hausdorff-Str. 2, 17489 Greifswald, Germany; 5 Department of Trauma and Orthopaedic Surgery, BG Klinikum Unfallkrankenhaus Berlin, Warener Straße 7, 12683 Berlin, Germany

**Keywords:** Ewing sarcoma, Chemotherapy, Cold physical plasma, Methotrexate, Cisplatin, Cancer, Cell biology, Oncology

## Abstract

Ewing's sarcoma (ES) is the second most common bone tumor in children and adolescents and is highly malignant. Although the new chemotherapy has significantly improved the survival rate for ES from about 10 to 75%, the survival rate for metastatic tumors remains around 30%. This treatment is often associated with various side effects that contribute to the suffering of the patients. Cold physical plasma (CPP), whether used alone or in combination with current chemotherapy, is considered a promising adjunctive tool in cancer treatment. This study aims to investigate the synergistic effects of CPP in combination with cytostatic chemotherapeutic agents that are not part of current ES therapy. Two different ES cell lines, RD-ES and A673, were treated with the determined IC_20_ concentrations of the chemotherapeutic agents cisplatin and methotrexate (MTX) in combination with CPP. The effects on population doubling, cell viability, and apoptotic processes within these cell lines were assessed. This combination therapy has led to a reduction of population doubling and cell viability, as well as an increase in apoptotic activity in cells compared to CPP monotherapy. The results of this study provide evidence that combining CPP with non-common chemotherapy drugs such as MTX and CIS in the treatment of ES enhances the anticancer effects of these drugs. These findings open up new possibilities for the effective use of these drugs against ES.

## Introduction

Ewing’s sarcoma (ES) is a rare but highly aggressive bone malignancy^1,2^. Its incidence is reported to be up to 1 case per 1.2 million population per year^3^. However, this number can vary depending on the ethnic population. Thus, African populations are significantly less affected by ES than Europeans^3–6^. The latest analyses of epidemiological data have shown an incidence of approximately 10 cases per million in white population groups^3^. Regardless of ethnic differences, the common feature is the manifestation of ES in adolescents at a mean age of 15 years^5,7–9^. Although ES typically occurs in bone tissue and primarily affects the axial skeleton, like pelvis and rips, and also proximal metaphysis of long bones, in about 20% of cases, it can also develop primarily in non-osseous tissue^9–12^. Cases of extraosseous ES are then diagnosed much more frequently in adults^13,14^.

The clinical diagnosis of ES can be challenging due to its non-specific symptoms, such as local pain that aggravates at night and swelling without an obvious cause. Pathologic fractures in the affected bones occur in about 10–15% of ES cases. The radiological analysis is much more meaningful than the diagnostic method. Multiple lytic bone lesions, Codman's triangle, and "onion peel" are typical radiological periosteal signs of proliferative response to bone malignancy^15,16^. As the disease progresses, other non-specific symptoms may appear, including fever, night sweats, fatigue, and weight loss^17^. Most laboratory diagnostics of the blood often show non-specific increases in inflammatory parameters and bone turnover, but an increased serum lactate dehydrogenase level correlates with this tumor burden and is, therefore, an important prognostic marker^15^. However, the most reliable diagnosis is made by identifying characteristic ES chromosomal translocations^18–20^.

Until the 1960s, the diagnosis of ES was fatal for approximately 90% of patients^21^. Several factors contribute to the prognosis of ES, such as the patient’s age and, histological characteristics, but the most important of these is the presence of metastasis at the time of diagnosis^22,23^. ES disease metastasis is found mostly in the lung, bone, or bone marrow^24^. With the introduction of chemotherapeutic agents in the treatment of ES, the survival rate of patients increased^25^. The current strategies against ES include combinations of chemotherapeutics such as doxorubicin, vincristine, and ifosfamide^26,27^ led to improvement in the prognosis of ES, raising the survival rates of up to 70%^18,26,28,29^. Other chemotherapeutics agents such as cisplatin and methotrexate have also been found to increase the prevalence of limb salvage surgery and to improve the 5-year survival rate to over 50% for bone sarcomas^30^, but those are not a part of the current treatment protocol for ES. Regardless of the positive influence on the survival rate of the patients, chemotherapy drugs have a number of side effects with high toxicity for many organ systems, such as kidneys, liver, heart, and nervous system, that remain a problem in the clinical setting^31–33^.

Looking for ways to solve this problem and to accelerate the arsenal against ES, cold physical plasma (CPP) has been suggested as a powerful tool in local anti-cancer therapy. Numerous studies have reported on the anti-oncological effect of CPP in many different types of cancer^34^, such as skin cancer^35,36^, breast cancer^37–39^, brain cancer^40,41^, and colon cancer^42–44^. On an experimental level, the inhibitory effects of CPP have also been demonstrated in the most common primary bone tumors, osteosarcoma and chondrosarcoma^45–49^, and also in ES^50^.

A strong inhibition of cell growth and viability of ES cells after CPP treatment has recently been reported and the advantages of combining CPP and the most commonly used chemotherapeutic agents in a clinically relevant in vitro setup^51^. The current study aims to evaluate whether CPP treatment leads to enhanced effectiveness of chemotherapeutic agents methotrexate (MTX) and cisplatin^52^ that have not yet been included in the list of "standard chemotherapy" against ES. Any opportunity to expand efficacy against ES is an advance that may impact patient care in ES therapy in the future.

## Materials and methods

### Ewing’s sarcoma cells

The Ewing sarcoma cell lines RD-ES (DSMZ-Deutsche Sammlung von Mikroorganismen und Zellkulturen, Braunschweig, Germany) and A673 (American Type Culture Collection, Manassa, VA, USA) were used in this study. Dulbecco’s modified Eagle’s medium (DMEM) and Roswell Park Memorial Institute 1640 medium (RPMI) were used for culturing the ES cells according to the producer's recommendation. DMEM contained 1.0 g/L glucose, 10% fetal bovine serum, 1 mM sodium pyruvate, and 1% penicillin/streptomycin, while RPMI 1640 contained 10% fetal bovine serum and 1% penicillin/streptomycin. The ES cell lines were grown under standardized conditions—(humidified atmosphere with 5% CO_2_ at 37 °C).

### Chemotherapeutic agents

MTX and CIS (both from Cayman Chemical, Ann Arbor, MI, USA) were prepared by dissolving the dry substance in DMSO (Carl Roth, Karlsruhe, Germany). The stock concentrations for the chemotherapeutic agents were as follows: MTX 10 mM and CIS 1 mM. These solutions were then diluted to the final concentrations using DMSO.

### Treatment with cold physical plasma

The kINPen^®^ med device (neoplas tools, Greifswald, Germany) was used for CPP treatment. The plasma flame is generated in a plasma jet that is suitable for manual use. Argon (Alphagaz 1 AIR LIQUIDE, Düsseldorf, Germany) is used as the carrier gas with a flow rate of 3.5 standard liters/minute (slm). The CPP application took place after the cells had been seeded in a 24-well plate with 2 × 10^4^ cells per 200 µL. The plasma flame was passed over this cell suspension with pendulum-like hand movements for different periods (5 s, 10 s, and 20 s). The control treatment of the cells was performed in the same way but without switching on the plasma device, i.e., only with the carrier gas argon.

### Application of chemotherapeutic agents

The addition of chemotherapeutics was done as previously described^54^. Briefly, or each cell line, 2 × 10^4^ cells per 200 µL of full medium were transferred to 6 wells of a 24-well plate. Subsequently, 800 µL of warm, fully-supplemented medium containing the corresponding cytostatic agent (MTX or CIS), dissolved at their determined IC_20_ values, was added to each well and incubated at 37 °C. As a control, a similar treatment with the carrier solution DMSO was carried out.

### Combination treatment with cold physical plasma and chemotherapeutic agents

Both cell lines were seeded separately with 2 × 10^4^ cells in 200 µL of full medium and directly exposed to CPP in the wells of a 24-well plate. After 5 s CPP exposure, 800 µL of warm, fully-supplemented medium containing the corresponding IC_20_ concentration of each cytostatic agent (MTX or CIS) was added to each well prior to incubation at 37 °C. Control cells were treated only with cytostatic agents.

### Quantification of the population doublings

After performing a single treatment (either 2.3. isolated 5 s CPP application; or 2.4. exposure to chemotherapeutic agents; or 2.5. combined treatment), cells were incubated over a 120 h period. The number of living cells was determined using the CASY cell counter and analyzer (Roche Applied Science, Mannheim, Germany). The population doublings were calculated using the following formula: PDL = PDL_0_ + 3.322 (logC_f_ − logC_i_).

### CellTiter-Blue cell viability assay

The CellTiter-Blue cell viability assay (Promega, Walldorf, Germany) was utilized according to the assay protocol to determine the cell viability of cells after being treated either only with a chemotherapeutic agent or after a combined treatment of CPP and chemotherapeutic agent.

After incubation for 24 h, the ES cells were treated with MTX or CIS in different concentrations from 10^–11^ to 10^–4^ M. The cytostatic drug preparations were performed by diluting to the desired concentrations in DMSO. The solutions were further diluted 1:100 in fully-supplemented medium before being administered to the cells. The cells were then incubated for 72 h at 37 °C before cell viability quantification with the CellTiter-Blue reagent.

As a second experiment, a total of 100 µL of cell suspension containing 1 × 10^4^ cells was seeded into 96-well plates and incubated for 24 h prior to the combination therapy. CPP treatment was carried out indirectly by transferring 200 µL of the full medium into the wells of a 24-well plate and treating each well with CPP for 5 s, 10 s, and 20 s. Then, 100 µL of the treated medium was added to the cells in the 96-well plates, followed by the addition of 100 µL of warm complete medium containing the cytostatic drug, and subsequently incubated at 37 °C for 72 h, followed by the determination of the cell viability using CellTiter-Blue reagent.

The CellTiter-Blue reagent was incubated for 1 h, and the formation of resorufin in living cells was detected using a multimode plate reader at 560 nm excitation and 590 nm emission wavelengths (TECAN, Männedorf, Switzerland). The fluorescence signals of the cells treated with the cytostatic drugs were then normalized to the signals of cells treated with DMSO (control) to determine their respective cell viability.

### TUNEL assay

A total of 5.0 × 10^4^ (24 h) and 2.0 × 10^4^ (48 h) from the A673 cells and 4.0 × 10^4^ (24 h) and 2.0 × 10^4^ (48 h) RD-ES were seeded in 100 µL. This cell suspension was transferred to a 96-well plate for the respective treatments. After a 5 s CPP treatment was performed indirectly by treating 200 µL of a full medium, 100 µL of the treated medium was added to the seeded cells. An argon treatment for 5 s served as control. In the next step, an IC_20_ concentration of MTX or CIS was added to the cells. As a control, a combination of an argon treatment and cytostatic drugs was performed. Controls with untreated cells (1 negative and 1 positive; nuclease treated) were included on each plate. A corresponding second plate was treated in parallel to normalize the absorption values to cell numbers. The TUNEL assay (R&D Systems, Minneapolis, MN, USA) was performed 24 h or 48 h after treatment according to the manufacturer’s protocol using the TECAN multimode plate reader. The relative TUNEL signals of cells treated with CPP, cytostatic or combination therapy were normalized to the mean relative TUNEL signals of cells treated with argon gas alone (control).

### Caspase assay

The CellEvent Caspase 3/7 green Detection Reagent (Thermo Fisher Scientific, Waltham, MA, USA) was used to detect apoptosis by performing the Caspase 3/7 assay. The detection reagent binds fluorescently to DNA, and DEVD peptide inhibits this binding. Upon activation of Caspases 3 and 7, the peptide is cleaved, allowing the binding to occur. The treatments were performed similarly to 2.8. After the incubation period (24 h and 48 h), the medium was carefully aspirated, and 100 µL of Caspase 3/7 detection solution was added to the wells of the 96-well plate. The plate was then incubated for 45 min at 37 °C. Following the 45-min incubation, fluorescence was measured using the TECAN multimode plate reader at 495 nm excitation and 535 nm emission wavelengths. The fluorescence per well was used to calculate the fluorescence per cell by deviding by the cell numbers determined using counting with the CASY device.. The relative Caspase 3/7 signals of cells treated with CPP, cytostatic, or combination therapy were normalized to the mean relative Caspase 3/7 signals of cells treated with argon gas only (control).

### DNA damage detection

A total of 1.0 × 10^5^ cells was seeded in 100 µL medium into a 96-well plate. The next day, cells were either treated with CPP followed by the addition of IC_20_ concentrations of CIS or MTX, or CPP, or cytostatic drugs alone, as described above (2.8). After 24 h incubation, the medium was discarded, and cells were washed carefully with DPBS. For fixation and permeabilization, cells were covered with ice-cold methanol for 30 min. After washing, cells were stained with PE-conjugated anti-ATM phospho and AF647-conjugated anti-H2A.X phospho antibodies (both Biolegend, Amsterdam, The Netherlands) for 30 min. Cells were washed again, and finally, 100 µL of PBS containing 4ʹ,6-diamidino-2-phenylindole (DAPI, 1 µM) were added to the cells to stain whole nuclei. Images were acquired using the high-content imaging system Operetta CLS (PerkinElmer, Hamburg, Germany) with a 20× objective, and quantification was performed using Harmony 4.9 software (PerkinElmer, Hamburg, Germany).

### Statistical analysis

For data analysis and visualization, GraphPad Prism Version 9.1.2 (GraphPad Software Inc., La Jolla, CA, USA) was used. The results of p ≤ 0.05 of at least three independent experiments were considered significant, and data were given as the mean ± SD. Differences were examined using an ANOVA test or t-test, as indicated in the figure captions.

## Results

### Impact of chemotherapeutic agents on cell viability and proliferation

**Figure 1:**
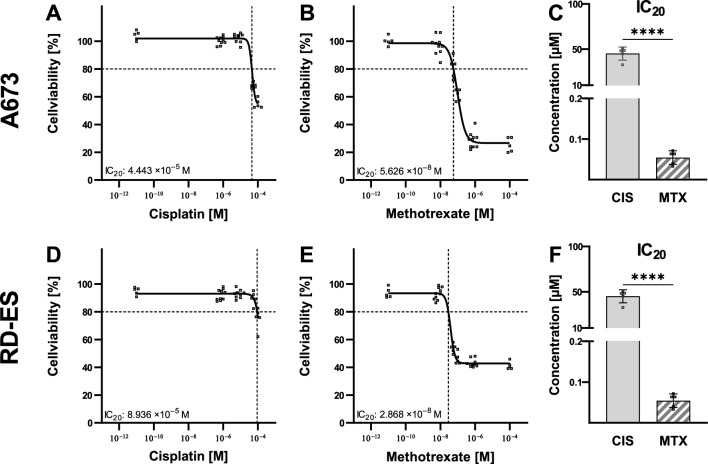
Inhibitory effect of cisplatin and methotrexate on Ewing’s sarcoma cells. The Ewing’s sarcoma cell lines A673 (**A**–**C**) and RD-ES (**D**–**F**) were treated with cisplatin and methotrexate (MTX). After 72 h, the cell viability was determined, and the IC_20_ was calculated. Individual values are shown with a dose–response curve and the IC_20_ calculated from this (**A**,**B**,**D**,**E**) as well as mean values of the calculated IC_20_ (**C**,**F**). The mean values were examined with the t-test for significant differences, which were presented as follows: ****p < 0.0001.

In order to investigate the influence of the chemotherapeutic agents CIS and MTX on ES cells A673 and RE-ED, these were incubated for 72 h with the chemotherapeutics in concentrations ranging from 10^–11^ M to 10^–4^ M. Cell viability (2.7) was determined after that. Both chemotherapeutic agents have inhibited the cell viability of A673 and RE-ED (Fig. 1). However, the chemotherapeutic agents had varying impacts on both ES cell lines. Using the maximum concentration of 100 µM of CIS, cell viability was reduced by approximately 50% in the cell line A673, and a 30% reduction in viability was observed in the cell line RD-ES. Using MTX in the same concentration, stronger inhibitory effects on the cell viability were achieved. For A673 cells, a reduction in cell viability of 80%, and for the RD-ES cell line, a reduction of 60% was shown. These effects and correlations have also been found by determination of the IC_20_ concentration of these chemotherapeutic agents. There were significant differences between the IC_20_ concentrations of CIS and MTX in both cell lines (p < 0.0001). IC_20_ of CIS was about 1000-fold higher than the IC_20_ of MTX (Fig. 1C,F).

### Impact of combination of cold physical plasma and chemotherapeutic agents

**Figure 2:**
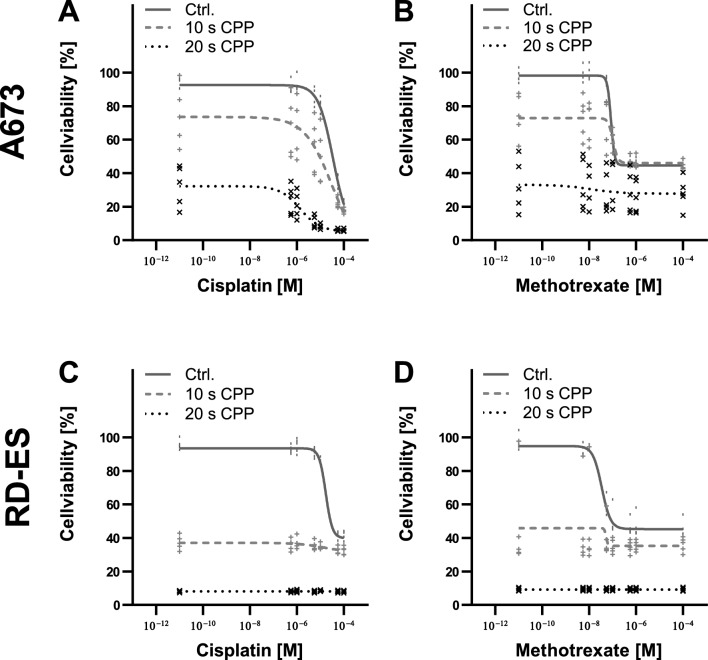
Effect of a combined treatment of ES cell lines with CPP and chemotherapeutics. The Ewings sarcoma cell lines A673 (**A**,**B**) and RD-ES (**C**,**D**) were treated with CPP combined with the chemotherapeutic agents cisplatin and methotrexate (MTX). Cell viability was determined after 72 h. Shown are individual values with dose–response curves.

**Figure 3:**
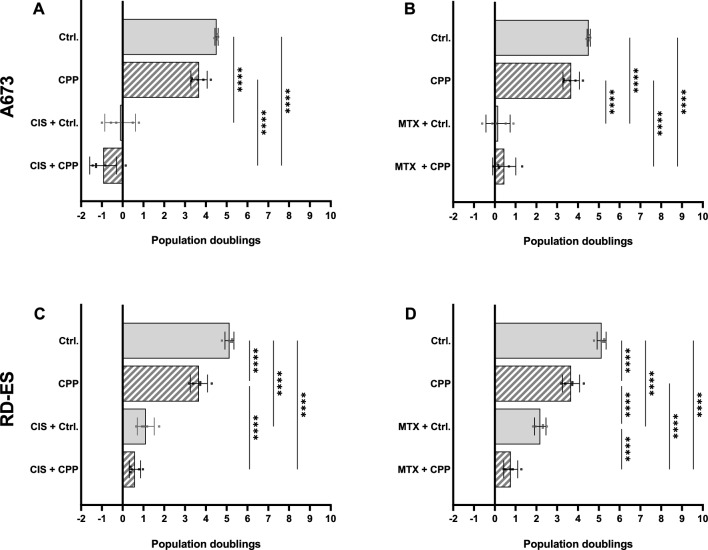
Effects of CPP treatment combined with chemotherapeutic agents on cell population doubling. A673 cells (**A**,**B**) and RD-ES cells (**C**,**D**) were treated with CPP and a combination of CPP and cytostatics. The number of living cells was determined after 120 h of incubation and the population of doublings was calculated. Mean values are shown. Significant differences were determined using ANOVA and Tukey's multiple comparisons test and are reported as follows (*p ≤ 0.05, **p ≤ 0.01, ***p ≤ 0.001, ****p ≤ 0.0001).

A combined treatment of the cell lines with CPP and MTX or CIS led to a stronger inhibitory effect on the cell viability. These effects were dependent on the treatment time with CPP (Fig. 2). A combination of chemotherapeutics and CPP treatment for 10 s led to a shift in the dose–response curve towards lower cell viability. In cell line A673, the 10 s CPP treatment in combination with CIS and MTX below the effective threshold led to a reduction in cell viability of over 20%. In general, the additive effects were significantly milder in this cell line. In contrast, the additive effects of CPP and chemotherapeutics agents were observed in the cell line RD-ES over the entire concentration range investigated. While a single CIS treatment of the RD-ES cells, even at a concentration of 100 µM, led to a reduction of about 80%, the combination with CPP achieved a reduction in cell viability of less than 40%. A combination of the chemotherapeutic agents with 20 s CPP led to a further reduction in cell viability in all cell lines. In the RD-ES cell line, the 20 s CPP treatment was already so effective below the threshold of the chemotherapeutics that the cells were almost completely obliterated. Thus, no further effect could be observed by increasing the concentration of chemotherapeutic agents. Experiments to cell population doubling confirm these results. A combination of CPP and chemotherapeutic agents can improve the effectiveness compared to treatment alone (Fig. 3). Thus, the population doublings of A673 cells were reduced from 4.4 to 3.7 after 5 s CPP treatment. Reductions to − 0.9^52^ or 0.67 (MTX) could be achieved through the combination of CPP and chemotherapeutics. The effects on the RD-ES cells were similar. CPP treatment alone reduced the population doublings from 5.13 to 3.66, the combination with CIS to 0.59, and the combination with MTX to 0.76.

### Cell apoptosis and DNA damage after combined treatment of cold physical plasma and chemotherapeutic agents

**Figure 4:**
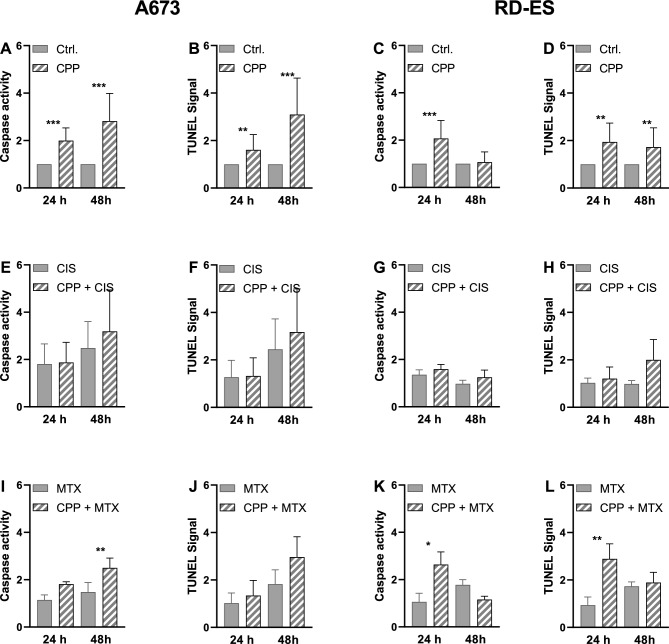
Apoptosis induction by the combination of CPP and cytostatic agents. To evaluate the apoptotic effect of combination therapy of the cell lines A673 and RD-ES Caspase 3/7 activity assay and TUNEL assay were used. The relative Caspase 3/7 signals and TUNEL signals of cells treated with CPP and combination therapy were normalized to the relative Caspase 3/7 signals and TUNEL signals of cells treated with Argon (control). The mean values ± SD are depicted in the graphs and were assessed for statistically significant differences using paired t-tests (*p ≤ 0.05, **p ≤ 0.01, ***p ≤ 0.001).

To examine the mechanism of cell death, apoptosis assays (caspase 3/7 activation, and TUNEL) were performed. The cells were treated with CPP for 5 s prior to cytostatic treatment at the determined IC_20_ (Fig. 4). In A673 cells, CPP treatment alone led to a significant increase in the caspase 3/7 activity and TUNEL signal both after 24 h and after 48 h compared to the control treatment (Fig. 4A,B). The combination of CPP and MTX treatment led to a significantly increased caspase 3/7 activity after 48 h (Fig. 4I,J). The CPP treatment of the RD-ES cells only increased the caspase 3/7 activity after 24 h. After 48 h, no significant increase in the signal could be detected. In the TUNEL assay, we could detect a significant increase in the signal both after 24 h and 48 h (Fig. 4C,D). A combination of CPP and MTX led to a significant increase in caspase activity and relative TUNEL signal after 24 h in RD-ES cells (Fig. 4K,L). The combination of CPP and CIS led to increased apoptotic signals in both cell lines;, however, this was not statistically significant (Fig. 4E–H).

**Figure 5:**
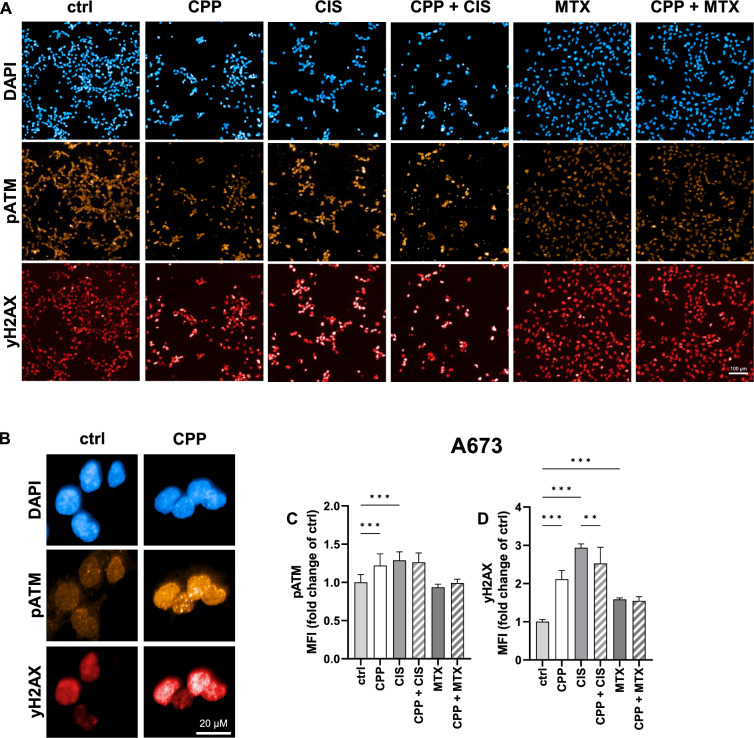
DNA damage marker formation upon CPP and chemotherapeutics treatment. A673 cells were stained 24 h after treatment with CPP, CIS, MTX, or combinations of these (**A**) to investigate the formation of nuclear pATM and yH2AX foci (**B**). Images were quantified by calculating the mean fluorescence intensity (MFI) of pATM (**C**) and yH2AX (**D**) in all detected nuclei of treated cells against untreated controls. The mean values ± SD are depicted in the graphs and were assessed for statistically significant differences using one-way ANOVA and Šídák's multiple comparisons test (*p ≤ 0.05, **p ≤ 0.01, ***p ≤ 0.001).

To further investigate toxic effects of CPP and cytostatic treatment, A673 cells were stained against the DNA damage markers pATM and yH2AX 24 h after IC_20_ single treatment or combination treatment (Fig. 5A). Formation of pATM or yH2AX foci in the nuclei (Fig. 5B) was quantified as mean fluorescence intensity (MFI) over all detected nuclei (Fig. 5C,D). In A673 cells, a significant increase of nuclear pATM was detected after CPP and CIS treatment, while MTX did not increase pATM (Fig. 5C). The combination of CPP and cytostatic drugs did not cause an additional increase in pATM. Significant formation of yH2AX foci was observed upon CPP as well as CIS and MTX single treatments (Fig. 5D). No additional increase of yH2AX but rather a decrease was visible in the case of combination treatments.

## Discussion

Since its introduction in the 1970s, chemotherapy has significantly improved the outcomes of patients with ES and is now an integral part of their treatment. A clear advantage over monotherapy has been shown in combination therapy with different drugs^53,54^. Besides the use of doxorubicin and vincristine, the use of ifosfamide, alone or in combination with etoposide, showed very good results in patients with ES and is currently part of the standard protocol for ES treatment^55–58^. The current 5-year survival rate varies and is 75% for patients with localized ES manifestations, around 50% for patients with isolated lung metastases, and less than 30% for people with primary metastases^59–61^.

The use of high doses of chemotherapeutic agents is associated with increasing their toxic effects on the patient^62,63^. A dose reduction with a constant effect of the chemotherapeutic agent would, therefore, be desirable. Such a dose reduction with the same effect on ES cell lines has been demonstrated with the combination of chemotherapeutic agents doxorubicin or vincristine and CPP^51^.

CPP treatment seems to be considered a possible anti-cancer tool that supports the effects of chemotherapy, not least because of its selective effect on tumor cells^39,64,65^. CPP treatment alone can significantly impact the viability of Ewing sarcoma cells by inducing apoptosis, disrupting cell proliferation, and inhibiting tumor growth^50^. While CPP treatment shows promising results in targeting cancer cells locally, its efficacy as a stand-alone therapy may not surpass that of chemotherapy in all cases. Despite its systemic nature, chemotherapy often remains a cornerstone of cancer treatment due to its ability to reach metastatic sites and eradicate disseminated tumor cells. The effect of the chemotherapeutic agents unfolds through binding to DNA and the production of ROS in the cells^66^. The action of CPP on various cancer cells unfolds in different ways—some of them, like apoptosis and DNA damage^46,50^, are very similar to the anti-oncogenic mechanism of action of chemotherapeutic agents. Thus, these two methods are able to complement their effects so that the dosage of the cytostatic can be reduced in order to achieve the desired effects^45^. A number of studies with different tumor entities have demonstrated the high efficiency of the combination of CPP and different chemotherapy agents^67–72^. The current study raised the question of whether the spectrum of anti-ES chemotherapy drugs can be expanded by combining them with CPP. MTX and CIS were frequently chosen for this in the treatment of other bone sarcomas. MTX is an effective therapeutic agent not only for treating many solid tumors but also autoimmune diseases. However, its use is limited by poor pharmacokinetics^73^. Among many chemotherapy drugs that are widely used for cancer, CIS is one of the most compelling ones^74^. It is effective against various types of cancer, including various sarcomas. Its mode of action has been linked to its ability to cross-link with the purine bases on DNA, causing damage to the cancer cells^66,75^. CPP also builds up its effect against cancer cells via similar mechanisms of action, so their combination can likely have a synergistic effect^46,50^.

This study showed that the isolated CPP treatment of the ES cells inhibited their proliferation capacity. A limitation of tumor treatment with CPP is its local intraoperative application. Disseminated individual cells or metastases cannot be addressed during tumor resection. Thus, CPP treatment only achieves a local effect. Therefore, it is of particular importance to consider CPP as a potential complementary therapeutic option in combination with established treatment modalities such as resection and systemic chemotherapy. Incorporating CPP into a multimodality treatment approach may more effectively target heterogeneous cancer cell populations, which could lead to better long-term outcomes for patients with Ewing's sarcoma. While CPP treatment alone is promising, combining it with chemotherapy is a rational strategy to maximize therapeutic efficacy and minimize adverse effects in the treatment of ES. The treatment of both cell lines A673 and RD-ES with CCP combined with MTX or CIS significantly increased their antiproliferative effect. The combination treatment also reduced cell viability, as these effects were CPP treatment time-dependent. Thus, a CPP treatment of 20 s CPP treatment even led to a complete reduction in cell viability of the ES cells. These results can be compared with the effects of the combined treatment of ES cells by CPP and the conventional chemotherapeutic agents typically used in clinical settings for these sarcomas, such as doxorubicin and vincristine^51^.

One of the main anti-oncogenic factors generated by CPP is the reactive species, which leads to apoptosis in cancer cells^71,72^. This mechanism of action has also been demonstrated specifically in bone cancer cells such as osteosarcoma and chondrosarcoma^48,49,76,77^, but also in ES cells^50^. The cytotoxic effect of CIS can also be explained by the inhibition of replication by cisplatin–DNA adducts and the induction of apoptosis^78^.

The combination of CPP treatment with chemotherapy may lead to increased tumor cell toxicity through several mechanisms. One possible mechanism is that the combination of CPP with cytostatic agents may lead to additive effects in cancer cells by enhancing drug uptake and increasing intracellular ROS induction^71^. In particular, CPP has been shown to increase the permeability of cell membranes, facilitating the entry of chemotherapeutic agents into cancer cells^50^. Furthermore, CPP treatment may synergize with specific chemotherapy drugs. For example, CPP-induced reactive oxygen and nitrogen species (ROS/RNS) production can interfere with DNA repair mechanisms, thereby sensitizing cancer cells to DNA-damaging agents like CIS. Similarly, CPP may modulate cellular signaling pathways involved in folate metabolism, potentiating the cytotoxic effects of MTX. Overall, the combination of CPP with chemotherapy can lead to increased toxicity of tumor cells by improving drug uptake, disrupting metabolic pathways that promote survival and achieving synergistic effects with specific chemotherapeutic agents. These effects ultimately lead to better treatment outcomes and can help to overcome the resistance mechanisms that often occur in cancer therapy. In-virto studies using mouse models were used to investigate the toxicity of individual CPP and chemotherapy treatments by analyzing biochemical markers in the liver and kidney. The results show that cold plasma significantly reduces the side effects of cytostatics (DOX)^79^. The combination treatment with CPP and cytostatics led to varying degrees of activation of apoptosis in each of the ES cell lines. In an in vitro study^80^, similar effects were found when combining CPP with low-dose CIS in head and neck squamous cell carcinomas cells, evaluating the cell viability, DNA damage, and apoptosis after this treatment^58^. The results of the current study indicate that concomitant treatment of CPP and chemotherapeutic drugs, like MTX and CIS, not included in the current ES treatment protocol, is promising in ES therapy.

Although the use of MTX and CIS in this study was effective against ES at the cellular level, these results cannot be prematurely extrapolated to the clinical treatment of ES. While there are reports of the use of CIS in the clinical setting, the use of CIS is part of a combination with other chemotherapeutic agents or as anecdotal reports^81^. The recommendation for the clinical use of chemotherapeutic agents does not only depend on the response of the specific cancer cell lines but also requires the consideration of many other factors, such as pharmacological properties, side effects, and interactions with other drugs in the treatment protocol. A number of preclinical and clinical studies are necessary to make such recommendations. However, an important finding from this study is the efficacy and interaction of chemotherapeutic agents and CPP in ES cells.

## Data Availability

The data that support the findings of this study are available from the corresponding author upon reasonable request.
